# Recent Advances in the Allergic Cross-Reactivity between Fungi and Foods

**DOI:** 10.1155/2022/7583400

**Published:** 2022-10-07

**Authors:** Haiyan Xing, Jianyong Wang, Yuemei Sun, Hongtian Wang

**Affiliations:** ^1^Department of Allergy, The Affiliated Yantai Yuhuangding Hospital of Qingdao University, Yantai, Shandong 264000, China; ^2^Department of Allergy, Beijing Shijitan Hospital, Capital Medical University, Beijing 100038, China; ^3^Department of Pediatric, The Affiliated Yantai Yuhuangding Hospital of Qingdao University, Yantai, Shandong 264000, China

## Abstract

Airborne fungi are one of the most ubiquitous kinds of inhalant allergens which can result in allergic diseases. Fungi tend to grow in warm and humid environments with regional and seasonal variations. Their nomenclature and taxonomy are related to the sensitization of immunoglobulin E (IgE). Allergic cross-reactivity among different fungal species appears to be widely existing. Fungus-related foods, such as edible mushrooms, mycoprotein, and fermented foods by fungi, can often induce to fungus food allergy syndrome (FFAS) by allergic cross-reactivity with airborne fungi. FFAS may involve one or more target organs, including the oral mucosa, the skin, the gastrointestinal and respiratory tracts, and the cardiovascular system, with various allergic symptoms ranging from oral allergy syndrome (OAS) to severe anaphylaxis. This article reviews the current knowledge on the field of allergic cross-reactivity between fungal allergens and related foods, as well as the diagnosis and treatment on FFAS.

## 1. Introduction

Among IgE-mediated food allergies, some are cross-reactivity between foods and inhaled allergens. In some cases, the presence of a respiratory allergy to an allergen with a shared epitope to food may lead to a clinically relevant cross-reactivity [1]. Plant-derived foods are predominantly involved by a prior sensitization to cross-reactive components present in pollen (grass, tree, and weeds), which can cause pollen food allergy syndrome (PFAS) [2]. Similarly, sensitization to fungi allergens via the respiratory tract and subsequent oral ingestion of cross-reactive proteins may lead to various food-allergic reactions, which can theoretically cause fungus food allergy syndrome (FFAS) [3]. At present, there are many studies on PFAS, while FFAS is still largely neglected in basic research as well as in clinical practice. This review summarizes characteristics and allergenic mechanisms of fungi, and particular attention is paid to allergic cross-reactivity between fungi and foods.

## 2. Characteristics and Classification of Fungi

Fungi are ubiquitous eukaryotic organisms with complete cell structure. The main components of fungal cell walls are mannoproteins, chitin, and *β*-glucans [4]. Fungi represent a distinct kingdom from animals, plants, and prokaryotes [5], without roots, stems, leaves, and chloroplasts. Fungi are dependent on organic nutrients provided by other organisms because they lack chlorophyll which is needed for photosynthetic processes [4]. As the common components in the atmosphere, they reproduce by generating spores through asexual or sexual processes [6]. Fungi are classified by domain, Kingdom, phylum, class, order, family, genus, and species like the classification methods of other organisms. Three phyla of fungi are specifically relevant to hypersensitivity disorders: *Zygomycota*, *Ascomycota*, and *Basidiomycota* [7], among which the *ascomycetes* including *Alternaria alternata*, *Aspergillus*, *Cladosporium*, and *Penicillium* are mainly allergen sources [8].

Fungi are closely related to human life; some fungi exist as edibles which are macrofungi with fruiting bodies [9], such as mushrooms, agaric, and tuckahoe. The fungi mainly involved in food fermentation are *ascomycetes* and *zygomycetes* [10, 11]. Some fungi are used for treating different ailments in traditional Chinese medicine including Ganoderma lucidum, Poria cocos, and Cordyceps sinensis [12]. Some mycotoxins or derivatives have been found for using as antibiotics (e.g. penicillin), antifungals (e.g. griseofulvin), and immunosuppressant agents (e.g. cyclosporine) [13]. Penicillin extracted from *Penicillium* is a well-known example of a mycotoxin used to reduce competition from bacteria [14].

Of more than 100,000 fungus species already described [15], the majority of them participate in the formation of normal human microbiome, while only a few are responsible for causing airway diseases [16]. Fungi have been described to cause adverse health effects in human beings through three specific mechanisms: direct infection of the host, elicitation of deregulated immune responses (allergic reactions), and toxic or irritant effects due to secondary metabolites [17–19]. Some fungi may colonize in the human body as human-related opportunistic pathogens [20]. Immunocompromised patients (diabetes, long-term immunosuppressive agents, HIV, etc.) are prone to fungal infections [21, 22], while atopic individuals are susceptible to fungal allergy [17]. In recent years, the prevalence of fungal sensitization tends to increase. The greatest risks in the case of fungal exposure are the development of respiratory diseases including fungal allergic rhinosinusitis, fungal allergic asthma, fungal allergic pneumonia, and allergic bronchopulmonary aspergillosis (ABPA) [23–25]. A study in German population analyzed fungal sensitization in patients with respiratory diseases over 20 years (1998–2017). It was shown that an increase in the rate of sensitization for almost all analyzed fungi was detected from the first decade of life to the second decade of life [7]. There is ample evidence demonstrating the unequivocal association among sensitization to *Alternaria* and the severity of asthma [26]. This paper focuses on allergic diseases caused by fungi, but sometimes it needs to distinguish from fungal infection or toxic diseases.

Medical terminology such as “mold sensitization” and “mold allergy” receive names familiar to the general public [7]. Mold describes a large group of different genera across the fungal phylum mainly growing in the form of hyphae, which then aggregate into web-like structures or mycelia [27]. Molds are multicellular fungi composed of hyphae and spores, while some unicellular fungi without hyphae or only pseudohyphae such as *yeast* and *Candida* are not included, which may still elicit allergic reactions [7]. *Candida* is one of the most prominent fungal members of the human microbiome with many species being commensals of the skin, gastrointestinal, and genitourinary tracts [28–30]. *Candida* is known as an opportunistic pathogen to cause infections in immunocompromised patients, such as thrush in infants and skin infections in diabetic patients [31, 32]. *Candida albicans* can serve as an allergen to cause allergic diseases in atopic individuals. Many studies have shown the association between immunoglobulin E (IgE) sensitization to *Candida albicans* and severity of atopic dermatitis [33]. Therefore, we use the more global term “fungi” throughout this article to describe FFAS.

## 3. Living Environment of Fungi

The distribution of fungi in nature varies with environmental conditions. Fungal spores are found as indoor and outdoor allergens. Outdoor fungi including *Alternaria*, *Cladosporium*, *Penicillium*, *Helminthosporium*, and *Aspergillus* [14] tend to grow on decaying vegetation and in the soil. Of which *Cladosporium* and *Alternaria* are two of the major genera of outdoor airborne fungi worldwide. Although there is also some variation, the most common taxa found in indoor environments include *Penicillium*, *Aspergillus*, *Curvularia*, and *Saccharomyces* [27]. Opportunistic fungi including *Candida*, *Malassezia*, and *dermatophytes* may colonize in the human body and induce infection or allergy under specific circumstances [34, 35].

The number of fungal spores in the atmosphere underlies seasonal as well as regional variations. In northern latitudes, outdoor fungal spore levels rise gradually with rainfall and increasing daily temperatures until they peak in the early fall [36]. A study in Western Thrace shows that the main spore season for fungal circulation in the atmosphere was observed from May to November. The highest concentration of *Alternaria* and *Cladosporium* was noticed in summer, with its peak in June for *Alternaria* and in July for *Cladosporium* [37].

Climatic factors such as temperature, rainfall, relative humidity, wind speed, and atmospheric pressure can influence the abundance of fungal species and their concentrations [38]. Months with high relative humidity and rainfall witness significant increase in fungal spore collection. Damp indoor environments including laundry rooms, bathrooms, storage rooms, and basements tend to have more elevated spore counts. Air conditioners and humidifiers are important reasons for the aggravation of indoor fungal pollution. Although optimal fungal growth requires high humidity, some xerophilic species of the genera *Alternaria* and *Cladosporium* are able to survive in a relative dry environment. During the day, outdoor humidity tends to peak in predawn hours, when hydrophilic fungi such as *ascospores* and *basidiospores* tend to reach their highest concentrations. However, *Alternaria* and *Cladosporium* generally peak in mid-afternoon during periods of low humidity [8, 39]. They are positively correlated with the daily average temperature and negatively correlated with the relative humidity [13, 37].

## 4. Mechanism of Sensitization to Fungal Allergens

In recent years, with the progress of molecular biology, genetics, and bioinformatics, specific immunoglobulin E (sIgE) levels in individuals sensitized to fungi appear to closely match their phylogenetic relationships which provide an opportunity to systematically look at allergic cross-reactivity among fungi [40]. Fungal spores and/or hyphae may cause allergic reactions after entering the human body through various ways, such as inhalation, ingestion, contact, and injection. It is well known that fungal cell wall components are widely conserved across fungi but absent in humans [4]. Many fungal allergens are localized in the cell wall of mature spores because they are convenient targets for immune recognition [41]. Fungal allergens, including proteases, protease activity receptor, glucans and membrane receptor, chitosanase, glycosidase, ribosome, mycotoxin, and volatile organic substances, can mainly cause type I, II, III, and IV allergic reactions [17, 42]. IgE-mediated type I hypersensitivity reaction is caused by exposure to fungal allergens and results in the activation of Th2 cells and Tfh cells. Th2 cells can promote the production of allergen-specific B cells by secreting IL-4, IL-5, IL-9, and IL-13, which further produce allergen-specific IgE antibodies [43]. IL-4 secreted by Tfh cells stimulates B lymphocytes to switch to plasma cells which produce large amounts of IgE antibody. In addition to Tfh cell-derived IL-4, IL-13-producing Tfh cells also promote IgE production [44, 45]. Subsequent exposure to the same allergens leads to an allergic reaction by degranulation of mast cells and basophiles which releases proinflammatory mediators and cytokines [46]. We mainly refer to IgE-mediated food allergy related to fungi in this paper, while some non-IgE-mediated or mixed reactions are not discussed in detail.

Fungi allergens typically show a considerable variability as a result of interstrain genomic differences, different culture conditions, and variable extraction procedures. Some allergen molecules are genera-specific or species-specific, while allergens with significant sequence homology are ubiquitous in some fungi which can cause cross-reactivity. Cross-reactivity is an immune-mediated phenomenon in which a specific antibody recognizes proteins homologous to the sensitizing allergen [47, 48]. In general, the closer the taxonomical relationship between species, the higher the degree of structural and immunological similarity between the allergens [49]. To date, a total of seventeen proteins are characterized as allergens in *Alternaria alternata*, of which Alt a1 is considered to be the only specific allergen component [50]. Alt a1 has been shown to have a very significant level of allergenic cross-reactivity with homologous fungal proteins from members of the *Pleosporaceae* family, including *Stemphylium*, *Ulocladium*, and *Curvularia* [51]. Alt a1 appears to be highly cross-reactive with several allergens of *Stemphylium* (Ste h1 and Steb1). Most of the other allergen components of *Alternaria* have homologues in the other three relevant mold genera in allergy: *Cladosporium*, *Penicillium*, and *Aspergillus* [33, 40, 50]. No specific major allergen components have been identified for *Cladosporium* allergies, while most registered allergens are cross-reactive minor allergens. For example, *Cladosporium* allergen Cla h8 has 75% sequence similarity with *Alternaria* allergen Alt a8 [33, 52]. Therefore, monosensitization to *Cladosporium* appears to be relatively rare [33]. Asp f1 is species-specific major allergen for *Aspergillus fumigatus*, which shows extensive sequence homology (95%) with *mitogillin* [40]. *Epicoccum* IgE cross-reactivity has been demonstrated between *E. nigrum*, *C. lunata*, *A. alternata*, *C. herbarum*, and *P. citrinum* [40].

**Figure 1:**
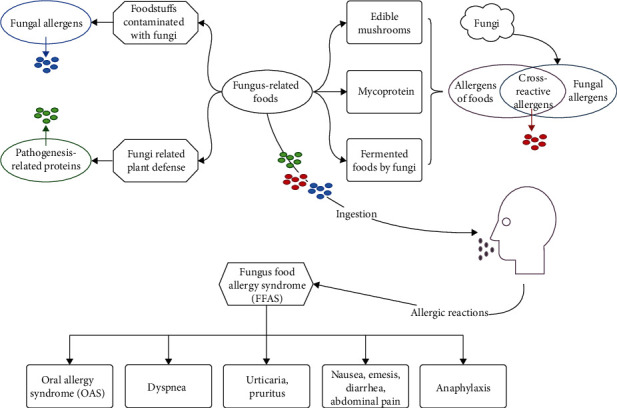
Schematic diagram of FFAS after ingestion of fungus-related foods.

Sensitization to fungi allergens and subsequent oral ingestion of cross-reactive fungal structures shared by fungi and foods can result in diverse patterns of allergic reactions. Fungus-related foods often induce to FFAS, and the existence of allergic cross-reactivity is an important mechanism of FFAS (Figure 1), which draws our attention to research deeply.

## 5. Allergic Cross-Reactivity between Fungi and Edible Mushrooms

Edible fungi are usually fruiting bodies produced by some *basidiomycetes* and *ascomycetes* [42]. Mushrooms are well-known examples of edible fungi which contain no cholesterol and are eaten as a good source of protein [53]. Mushroom spores exposed to the air are known as inhalation allergens [54]. A case report of 32-year-old woman with allergic asthma associated with exposure to mushroom spores was published by Branicka et al. in 2021 [55]. She developed symptoms of bronchial asthma during work in oyster mushroom farm, while the symptoms disappeared after leaving the workplace.

There are some described cases of mushroom-related allergy by ingestion; the symptoms are more diverse, such as OAS, urticaria, abdominal pain, vomiting, dyspnea, angina pectoris, myocardial infarction, and severe systemic allergic reactions [56, 57]. A few probably present a primary sensitization to mushrooms [56], and most of them are due to cross-reactivity with airborne fungal homolog allergens. Dauby et al. [58] reported the first case of OAS with uncooked mushroom in a patient allergic to molds. The patient with a history of allergic rhinitis complained of immediate lip, palate, and throat itching with the ingestion of raw mushroom. Heat labile mushroom proteins of 43 kD and 67 kD molecular weight range seemed to cross-react with aeroallergens from molds. Another study showed associations between allergenicity to airborne molds (*A. alternata* and *C. herbarum*) and food allergies, namely to mushrooms and spinach, which was referred to as “Alternaria spinach syndrome (ASY)” [59]. Continuing in this direction, further immunoblot/inhibition assays demonstrated the 30kD protein present in spinach and mushroom extracts and had a molecular weight similar to the major allergens of *Alternaria alternata* (Alt a1) and *Cladosporium herbarum* (Cla h1) [60]. In recent years, new cross-reactive proteins have been gradually found. Gabriel et al. [61] reported a patient who developed episodes of generalized urticaria and systemic anaphylactic shock immediately after ingesting mushrooms due to a prior sensitization to molds. A manganese-dependent superoxide dismutase (MnSOD) and a NADP-dependent mannitol dehydrogenase (MtDH) from Agaricus bisporus mushroom were identified as specific IgE-binding proteins. Cross-reactivity between *A. bisporus* MnSOD and mold aeroallergens was confirmed [61]. Betancor et al. [62] found a cross-reactive protein of about 36 kD which was identified as a member of the porin family both from button mushroom (*A bisporus*) and from a mold (*A alternata*). Ribosomal proteins S8 and S15a were identified as cross-reactive mushroom allergens, while they were not homologous to any reported fungal ribosomal protein aeroallergens [63].

In fact, knowledge of allergenic proteins that cause recognized clinically relevant cross-reactivity between fungi and edible mushrooms is still limited, and research in this field is needed to identify the causative allergens.

## 6. Allergic Cross-Reactivity between Fungi and Mycoprotein

Mycoprotein refers to the protein-rich food obtained from filamentous fungal biomass which can be used as an alternative to meat for human consumption [64]. Quorn is the trade name for a line of foods made with mycoprotein, which springs from the *fungus Fusarium venenatum* [65]. Mycoprotein, sold as Quorn, was developed in the United Kingdom (UK) and originally marketed there in 1985. Quorn-brand foods can serve as nutritious substitutes for meat products which are rich in essential amino acids (EAAs) and low in fat, cholesterol, sodium, and sugar [66].

Since the introduction of Quorn entered the UK marketplace, there have been complaints from consumers reporting numerous adverse reactions including urticaria and pruritus; swelling of the throat, tongue, mouth, or lips; breathing difficulties; anaphylaxis; and nausea, emesis, diarrhea, and abdominal cramps. The fact that 72.4% of allergic reactions and 67.6% of gastrointestinal reactions occurred on an individual's first exposure to a Quorn food suggests a cross-allergenicity with other antigens [67]. Hoff et al. [68] described the case of an asthmatic patient who had an acute attack of asthma with urticaria/angioedema after ingestion of a mycoprotein food product. These symptoms probably were the result of allergic cross-reactivity between the mycoprotein derived from *F venenatum* and the 60S acidic ribosomal protein P2, which was identified as allergen Fus c1 from *Fusarium culmorum* and also described as allergen for the molds *C herbarum*, *A fumigatus*, and *A alternata*. Thus, patients who are allergic to fungi may react adversely to mycoprotein because allergenic determinants are shared between them. It is unknown whether the gastrointestinal symptoms after consumption of Quorn products were caused by IgE or non–IgE-mediated allergic reactions or sometimes mediated by a nonimmunological mechanism, which need to be further studied [67]. However, adverse reactions of any kind to mycoprotein are rare, and for the vast majority of individuals, mycoprotein represents a safe foodstuff [66].

Nowadays, mycoprotein is produced at a large-scale using fermentation methods and commercially available in the USA, Europe, Asia, and Australia [69, 70]. However, edible fungal proteins obtained mainly from *Fusarium venenatum* and *Aspergillus*, which are not mainstream edible fungi in China. The acceptance in the food supply of this nonessential ingredient deserves reconsideration.

## 7. Allergic Cross-Reactivity between Fungi and Fermented Foods by Fungi

Fungi have been consumed for many years by humans as components of fermented foods, aiming to prolong the shelf-life, reduce the volume, shorten the cooking time, and improve the nutritive value of the food [64]. *Penicillium roqueforti* and *Penicillium camemberti* are essential to produce blue and soft-ripened cheese. *Monascus purpureus* is used in the production of red yeast rice. *Aspergillus oryzae* and *Rhizopus* species ferment soybeans to produce hamanatto, miso, tempeh, and shoyu [5, 64]. Fungi utilized in the fermentation process can serve as allergens and result in allergic reactions after ingestion.

*Yeast* species of the genus *Saccharomyces* are used in fermentation processes to produce alcoholic beverages. Wine is made from fermented grape juice, and beer is brewed from cereal grains fermentation by *yeasts*. Hypersensitivity reactions to beer or wine are rare and have been mainly attributed to grains or grapes [71]. However, *yeasts* should be considered as possible ingestive allergens in mold-allergic patients. A case with a clustered respiratory IgE sensitization to fungi (*Alternaria alternata*, *Cladosporium herbarum*, *Aspergillus fumigatus*, and *Penicillium notatum*) and *yeast* (*Saccharomyces cerevisiae*) developed multiple anaphylactic reactions after ingesting beer, red wine, sauces, and pasta yeast sauces containing cross-reactive fungal allergens [72]. All the serious allergic reactions took place in autumn when the concentration of molds in the local air was generally high. The inhalative exposure to mold aeroallergens in autumn might have increased the patient's sensitivity to the *yeast* ingested. *Yeast* sensitization has also been described as the cause of allergy to beer, cider, and wine in another report [73], and symptoms included throat and facial itching accompanied by mild wheeze and severe urticaria. German-Sanchez et al. [74] reported a case of beer and wine allergy caused by *Saccharomyces cerevisiae* allergy. She had a previous history of respiratory allergy due to multiple fungi, and subsequently she developed anaphylactic reactions after drinking beer and wine. As is known to us, heating could destroy some relevant food allergens. Some cooked fruits and vegetables typically do not elicit allergic symptoms in PFAS, because cross-reactive epitopes could be denatured with heating [75]. Patients who drank heated alcoholic beverages and baked bread would not cause allergic symptoms, suggesting that some *Saccharomyces cerevisiae* allergens could be inactivated with heat. More research will be necessary in the future to identify and characterize allergenic proteins of *Saccharomyces*.

Allergic reactions caused by other fermented foods such as fermented sausage have also been reported [76]. Three patients suffered labial angioedema and oropharyngeal pruritus after eating a cured, dry catalonian sausage of pork meat fermented with *Penicillium chrysogenum blanc*, while none of the them presented any symptoms after ingestion of pork meat or spices. IgE-mediated mechanism was demonstrated, and the IgE-reactivity bands were different in each. Sensitization to different allergen components might induce different clinical symptoms.

In addition, foods may contain a high histamine content such as fermented cheeses with fungi, which can trigger nonimmune-mediated food intolerances [77]. These non-IgE-mediated or mixed reactions of fermented foods allergy related to fungi are also existed and deserve attention.

## 8. Allergic Reactions between Fungi and Foodstuffs Contaminated with Fungi

The decomposing characteristics of fungi are common phenomena causing the spoilage of foodstuffs [14]. Food allergy to fungi occurs not only after ingestion of traditional fungus-related foods but also after accidental contaminated foods with fungi. Fungal contamination of fruits, vegetables, and other foodstuffs probably make them important sources of hazardous mycotoxins and fungal allergens. The presence of mycotoxins in foodstuffs might be used as a direct indicator of fungal contamination and thus of food quality and safety for human consumption [78]. Patients who apparently were not sensitized to these foods in the past may suffer from allergic reactions with exposure to fungi allergens by ingestion of contaminated foods.

Bennett and Collins [79] reported an unusual case of fatal anaphylaxis due to heavy mold contamination of a pancake mix with molds (*Penicillium*, *Fusarium, Mucor*, and *Aspergillus*), in a 19-year-old white male allergic to molds, pets, and penicillin. The decedent suffered short of breath and cardiopulmonary arrest after ingestion of pancakes with severe mold pollution.

It is also possible that bee pollen supplements, to be contaminated with fungi such as *Aspergillus* and *Cladosporium*, might cause severe allergic reactions in patients sensitized to these molds [80, 81].

Another research recruited 29 *Alternaria*-allergic patients with asthma but no food allergies. Some patients experienced allergic respiratory symptoms when they used their teeth to crack open the sunflower seed shells due to the inhalation of fungal proteins in the contaminated sunflower seeds. Sunflower Seed–Fungus Syndrome was involved in to describe it. Alt a1 is the main allergen from *Alternaria* isolated from sunflower seed shells. Immunoblotting inhibition demonstrated that specific IgE against *Alternaria* proteins have cross-reactivity with proteins from the other contaminated fungal species including *Aspergillus*, *Cladosporium*, *Penicillium*, and *Rhizopus* [82].

## 9. Fungi-Related Plant Defense and Allergy

Due to the constant threat of attack from various types of pathogens, such as viruses, bacteria, and fungi, plants exhibit defense properties and express so-called pathogenesis-related proteins (PR-proteins) [83]. The majority of allergen components involved in cross-reactivity between aeroallergens and plant origin foods belong to the group of PR-proteins. Thaumatin-like proteins (TLPs) are plant defense-related proteins of the PR-5 family that have antifungal activity [84, 85].

The genus *Alternaria* is a common kind of fungi including many saprophytic, endophytic, or even pathogenic species in nature, which can infect a wide variety of fruits and vegetables [86]. The studies found that Alt a1 was detected in kiwifruits infected with *Alternaria* despite without visible signs of infection, which induced the expression of the kiwi PR5-TLP (known as Act d2) [50, 87]. Both Alt a1 and PR5-TLP localized in the kiwi pulp and interacted with each other. Alt a1 inhibited the antipathogenic activity of the PR5 proteins and characterized as an enzymatic inhibitor of the PR5 family. A cosensitization phenomenon between Alt a1 and PR5-TLP was caused by the ingestion of kiwifruits infected with *Alternaria* but apparently in good conditions. This effect was not limited to kiwi PR5 only, while PR5 from other fruits, such as peach and banana, also interacted with Alt a1. Thus, *Alternaria*-allergic patients may experience an allergic crisis after consumption infected fruits with *Alternaria*.

Another research showed the sensitization to fungi occurred in 30% patients of atopic dermatitis, who suffered more hypersensitivity reactions to nuts (walnuts, peanuts) and sea fish. The authors speculated that nuts and fish might have some protective antifungal effects [88].

## 10. Diagnosis and Treatment of FFAS

The association between primary IgE sensitization with respiratory symptoms to fungi allergens and food allergy due to cross-reactive allergen components is important to assess in allergy practice. It is now generally accepted that correct diagnosis of FFAS should be evaluated within the framework of a patient's clinical history though there is oftentimes more challenging and difficulties. Atopic patients with a history of inhalation fungal allergy trigger allergic reactions during or just following ingestion of fungus-related food, which apparently were not sensitized in the past. Clinical suspicion of the FFAS is based on prick or intradermal skin tests in vivo diagnosis, the determination of fungi allergen-specific IgE antibodies in vitro diagnosis. When available, component-resolved diagnostics is a reliable instrument in the diagnosis of FFAS, as it provides profiles based on the cross-reactive proteins. Eventually, the double-blind, placebo-controlled food challenge (DBPCFC), as known as the food provocative test, remains the gold standard in diagnosis of food allergy, which should apply equally to FFAS [89].

Management of FFAS substantially relies on allergen avoidance and emergency treatment to allergic reactions. Allergen avoidance is the safest strategy at present including decreased exposure to fungal allergens and dietary avoidance. Dietary avoidance should be individualized recommended only if food allergy due to cross-reactivity is based on a clear history or on a clinical observation after oral provocation tests [90]. Of note, atopic individuals must not be put on diet according to their sensitization pattern alone [91]. Antihistamines blocking specific H1 receptors could be effective in the case of itching and urticarial [77]. Adrenaline should be administered early in cases of anaphylaxis due to accidental ingestion of the culprit food, which is crucial to prevent the fatal outcome of anaphylactic reactions [77]. Allergen immunotherapy (AIT) is currently the cornerstone of IgE-mediated allergy treatment, which has been used for over a century [92]. The aim of AIT is to alter the allergic response to allergens so that the patients become desensitized or, possibly, tolerant to the fungus-related foods. Food allergen-specific therapies have not been applied in FFAS. We speculate that the complexity of the relationship between fungi and foods prevent the application of AIT. The evidence for AIT modifying the underlying inhalant allergy to be efficacious to treat the associated cross-reactivity is contradictory. Beneficial effects of AIT on PFAS have been described. A recent study found that in patients with PFAS, the application of pollen extract subcutaneous AIT could not only improve the tolerance to pollens, but also reduced the symptoms of patients with food allergies [93]. However, these results cannot be reproduced in other studies. There is no clear evidence that pollen AIT is helpful to cross-reactive foods in OAS [91, 94]. AIT in birch-apple syndrome showed a limited beneficial effect on apple allergy [95, 96]. There are few studies on the fungal AIT in FFAS, the lack of standardized fungal allergens is one of the reasons [97]. However, more prospective, large-scale, double-blind, placebo-controlled studies are needed to further evaluate the efficacy of fungal allergen specific AIT in FFAS. Anti-IgE monoclonal antibody such as Omalizumab could be proposed for treating food allergy [98]. It should be a good choice in FFAS which need further study.

Antifungal agents including azoles, polyenes, Echinocandins, allylamines, and antimetabolites are drugs for the treatment of fungal infection [99, 100]. ABPA is a complex allergic disorder caused by immune reactions against the *Aspergillus fumigatus*. Antifungal triazoles such as itraconazole and voriconazole are used in the treatment against ABPA which could decrease the fungal burden [101, 102]. Generally speaking, antifungal agents play a limited role in fungal allergic diseases. At present, there is no report of antifungal therapy in FFAS.

## 11. Conclusions

Fungi widely live in nature and are one of the main airborne allergens. Fungus-related foods can exist as macrofungi with fruiting bodies, mycoprotein, fermented, and contaminated foods with fungi. Allergic reactions can occur during or after ingestion of fungus-related foods, and allergic cross-reactivity may be the main pathogenic mechanism. We mainly pay attention to IgE-mediated fungus-related foods allergy, while non-IgE-mediated route like FPIES (Food Protein-Induced Enterocolitis Syndrome) or mixed reactions for these foods are not discussed in detail. In fact, knowledge of allergenic proteins that cause recognized clinically relevant cross-reactivity between fungi and foods is still limited, and research in this field is needed to identify the causative allergens and to understand the immunological events that take place. There are limitations for insufficient studies with large samples in this area, and further research is needed to perform in the future.
